# Hormonal and biochemical changes in female *Proechimys guyannensis*, an animal model of resistance to pilocarpine-induced status epilepticus

**DOI:** 10.1038/s41598-020-77879-1

**Published:** 2020-12-02

**Authors:** Viviam Sanabria, Simone Bittencourt, Sandra R. Perosa, Tomás de la Rosa, Maria da Graça Naffah-Mazzacoratti, Monica L. Andersen, Sergio Tufik, Esper A. Cavalheiro, Débora Amado

**Affiliations:** 1 Department of Neurology and Neurosurgery, Universidade Federal de São Paulo (UNIFESP), Rua Pedro de Toledo, 669, São Paulo, SP Brazil; 2 Department of Psychobiology, Universidade Federal de São Paulo (UNIFESP), Rua Botucatu, 826, São Paulo, SP Brazil

**Keywords:** Neuroscience, Physiology, Zoology

## Abstract

The Amazon rodent *Proechimys guyannensis* is widely studied for hosting various pathogens, though rarely getting sick. Previous studies on male *Proechimys* have revealed an endogenous resistance to epilepsy. Here, we assess in female *Proechimys,* whether sex hormones and biochemical aspects can interfere with the induction of status epilepticus (SE). The lithium-pilocarpine ramp-up protocol was used to induce SE, and blood sera were collected at 30 and 90 min after SE, alongside brains, for biochemical, western blot and immunohistochemical analyses. Results from non-ovariectomised (NOVX) *Proechimys* were compared to ovariectomised (OVX) animals. Data from female Wistars were used as a positive control of SE inductions. SE latency was similar in NOVX, OVX, and female Wistars groups. However, the pilocarpine dose required to induce SE in *Proechimys* was higher (25- to 50-folds more). Despite a higher dose, *Proechimys* did not show strong SE like Wistars; they only reached stage 2 of the Racine scale. These data suggest that female *Proechimys* are resistant to SE induction. Glucose and progesterone levels increased at 30 min and returned to normal at 90 min after SE. A relevant fact because in humans and rodents, SE leads to hypoglycaemia after 30 min of SE and does not return to normal levels in a short time, a typical adverse effect of SE. In OVX animals, a decrease in GABAergic receptors within 90 min of SE may suggest that ovariectomy produces changes in the hippocampus, including a certain vulnerability to seizures. We speculate that progesterone and glucose increases form part of the compensatory mechanisms that provide resistance in *Proechimys* against SE induction.

## Introduction

Status epilepticus (SE) a condition characterised by is a prolonged seizure activity resulting from the failure of mechanisms responsible for ending seizures or from mechanisms that lead to seizures with a prolonged duration^1^. In humans and experimental animal models, sustained SE lead to neuronal loss in selective regions of the brain that can cause epilepsy or death.

The transition to SE can be preceded by biochemical changes and the gradual development of pathological brain electrical activity characterised by the involvement of an epileptogenic focus usually located in the hippocampus. Studies have shown that susceptibility to SE can be analysed by measuring biochemical and brain dynamics (i.e., neurochemical and electrical activities)^2,3^.

Susceptibility to SE in women is related to hormonal fluctuations during the menstrual cycle phases^4^. Traditionally, in the follicular phase, oestrogen is produced, a hormone classified as a proconvulsant as it reduces the convulsive threshold. In contrast, in the luteal phase, progesterone, a hormone considered an anticonvulsant agent due to its neuroprotective role, reaches high levels^4,5^. In rodent brain, oestradiol decreases gamma-aminobutyric acid (GABA)ergic inhibition^6^, whereas progesterone, via allopregnanolone, binds to GABA receptors and increases postsynaptic GABAergic effects^7^. As such, susceptibility to SE induced by pilocarpine in female rodents changes according to the phases of the oestrous cycle^8^. Other studies have demonstrated the neuroprotective role of ovarian hormones against induced seizures, which can be observed by comparing non-castrated with castrated females^9^.

*Proechimys guyannensis* (*P. guyannensis*) is a wild Amazon rodent^10^, commonly known as a natural host of infectious agents, although rarely getting sick^11^. In 2001, it gained considerable attention in neuroscience due to its striking resistance to epileptogenesis^12,13^. The reason for this resistance is not yet known. To date, until this study, neurological studies have been carried out only in males, due to variations caused by the female reproductive cycle.

Recently, we published a study on oestrous cycle of (*P. guyannensis*)^14^. Now we are moving forward with studies on possible hormonal interferences in the innate resistance to SE. In biomedical research, it is crucial to examine both sexes to ensure the study is translational^15,16^.

Therefore, the aim of this study was to induce SE in female *Proechimys* using the lithium-pilocarpine ramp-up protocol and analyse the latency to reach SE, the intensity of SE, and the hormonal and biochemical changes.

Here, we provide information on castrated and non-castrated females with biochemical and hormonal analyses (progesterone, oestrogen, glucose, cholesterol [total and fractions], GGT, T3, T4, sodium, creatinine, and urea); as well as immuno-blotting and immunohistochemical analyses in hippocampus for: glutamate (mGluR2/3), GABA (GABA_B_), parvalbumin, progesterone, oestrogen alpha (ER-alpha), and oestrogen beta (ER-beta) receptors. Castrated and non-castrated control groups were also analysed.

Female *Proechimys* showed resistance to SE induction with glucose and progesterone, indicating a possible key role in this resistance. Ovariectomy showed a decrease in GABAergic receptors after long exposure to SE (90 min), which may point to the fragility of these neurons during seizures and, thus greater hippocampal susceptibiliy to seizures, compared to non-castrated animals. Other considerations are highlighted in the results and discussion sections below.

## Results

### High doses of pilocarpine were required for female *Proechimys* to reach SE

**Figure 1:**
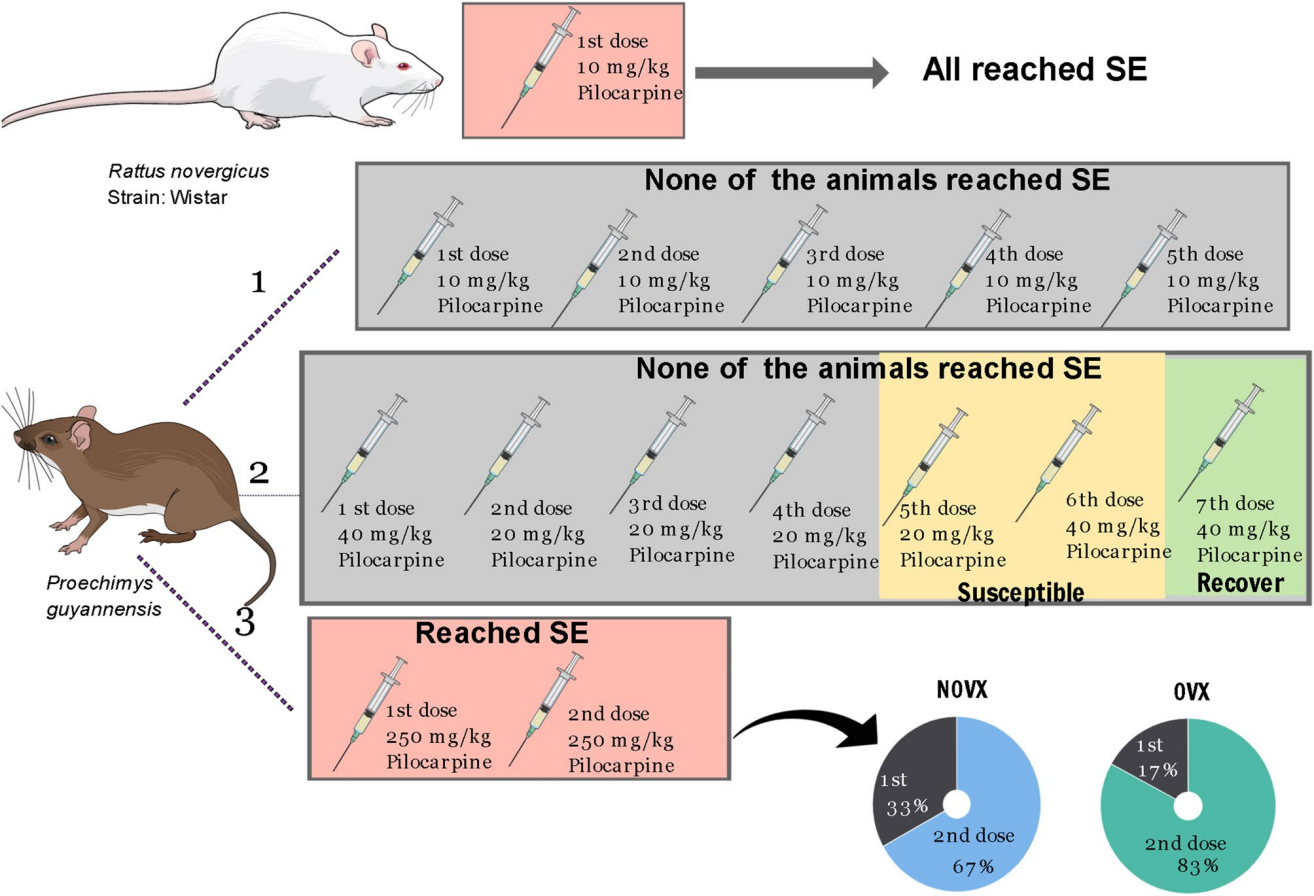
A schematic of the experimental design and the timeline of pilocarpine doses. Status epilepticus (SE) was first induced in female Wistar rats through graded intraperitoneal (ip) injections of pilocarpine, with the first dose of 10 mg/kg (ip). Wistar rats reached SE. *Proechimys* reached SE in the third evolution (3) of the lithium-pilocarpine ramp-up protocol with a dose of 250 mg/kg (ip). Among NOVX animals, 33% reached SE with the first dose and 67% with second dose (250 mg/kg). Among OVX animals, 17% reached SE with the first dose and 83% with the second dose (250 mg/kg). NOVX: Non-ovariectomised animals; OVX: Ovariectomised animals.

In female *Proechimys*, an initial dose of 10 mg/kg of pilocarpine was administered, followed by 10 mg/kg every 30 min for up to five doses of pilocarpine, as described in the lithium-pilocarpine ramp-up protocol^17^. As the initial dose was not effective, we followed certain steps until reaching the ideal dose of pilocarpine as described in Supplementary Information A and schematic Fig. 1.

The amount of pilocarpine, latency to reach SE and the intensity of SE in female *Proechimys* were compared with female Wistar rats used as positive control. The mean SE latency in female *Proechimys* (29.83 ± 15.05 min) was no longer than that in female Wistar rats (28.30 ± 11.84 min). However, while Wistar rats easily reached SE with a 10 mg dose of pilocarpine, *Proechimys* only reached SE after high doses of pilocarpine (250–500 mg) (Fig. 1).

With regards to the SE development according to Racine's scale^18^, female *Proechimys* showed similar patterns as those described for male *Proechimys* with abnormal behaviours typical of Racine stage 2, i.e., all four limbs on the floor, extended digits, mastication and head nodding, but no evolution to further stages^12,13^.

### The mean latency to reach SE was similar for NOVX and OVX groups

Of the 12 non-ovariectomised (NOVX) rodents, 4 (33%) developed SE after the first pilocarpine dose (250 mg/kg, ip), whereas the remaining 8 (67%) rodents converted to SE after the second dose. The mean latency of NOVX was 29.83 ± 15.05 min (mean ± SD). Of the 12 ovariectomised (OVX) rodents subjected to pilocarpine, 2 (17%) and 10 (83%) animals developed SE after the first and second dose, respectively, with a mean SE latency of 34.00 ± 9.83 min. The seizure latencies between OVX and NOVX animals were not significantly different. Based on the Racine scale, *Proechimys* OVX and NOVX reached a score of 2.

### *Proechimys* submitted to SE showed high blood levels of glucose and progesterone

**Figure 2:**
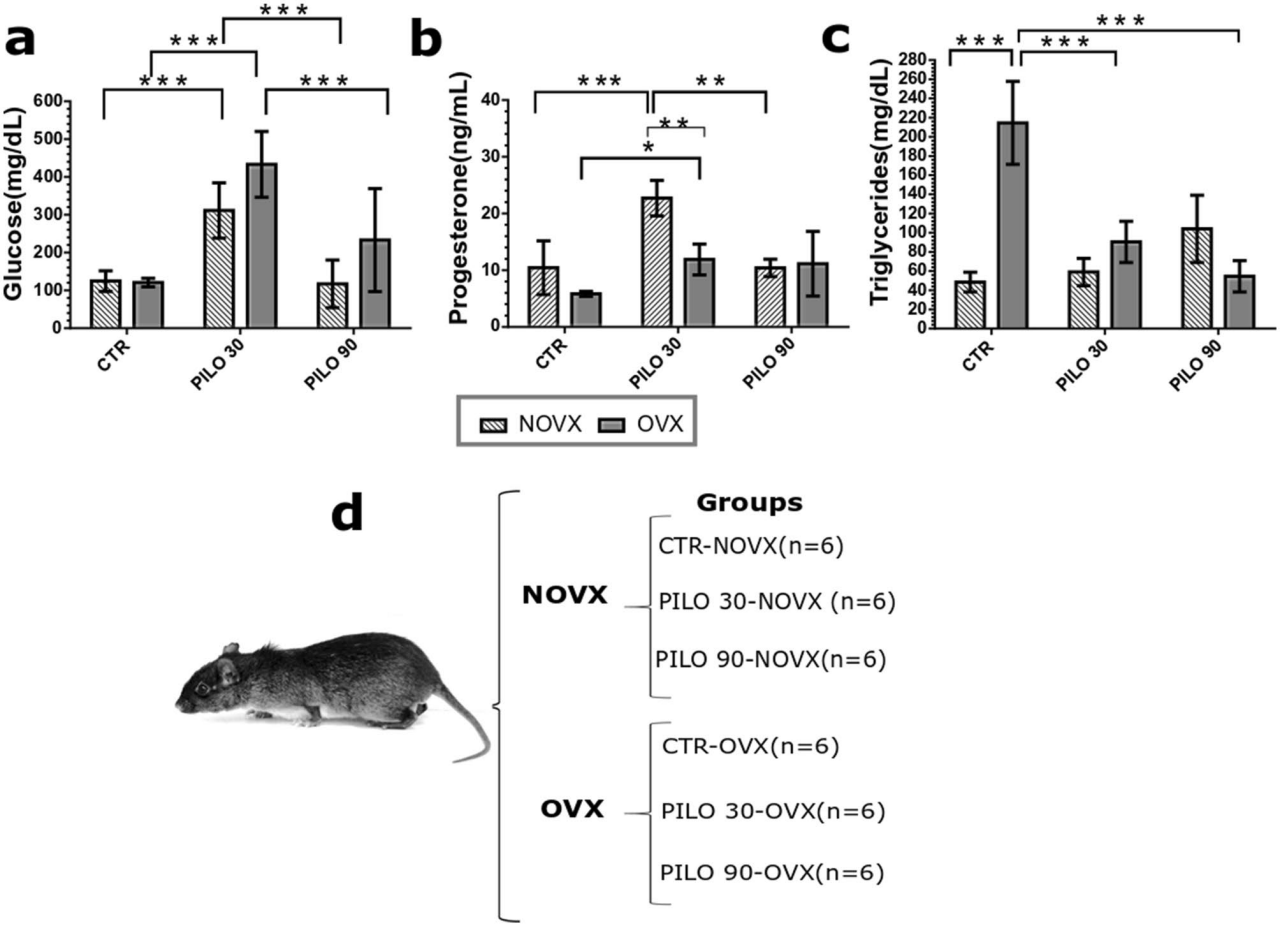
Effects of pilocarpine administration on glucose levels in *Proechimys*, an animal model of innate resistance to epilepsy. Experiments were performed in ovariectomised and non-ovariectomised animals. At 30 and 90 min after SE induction, blood serum was evaluated and changes in glucose, progesterone, and triglyceride levels were determined. (**a**) Serum glucose levels are presented in mg/dL. (**b**) Serum progesterone levels are presented in ng/mL. (**c**) Serum triglyceride levels are presented in mg/dL. (**d**) Group distribution of *P. guyannensis* in the present study. NOVX: Non-ovariectomised animals; OVX: Ovariectomised animals; CTR: Control; PILO 30: Data obtained 30 min after SE induction; PILO 90: Data obtained 90 min after SE induction. Data are presented as mean ± SD. Levels marked with asterisk (*) indicate significant differences: **p < 0.01, ***p < 0.001.

Glucose levels in NOVX and OVX animals were significantly increased (*p* < 0.001) at 30 min after SE (311.00 ± 72.85 and 433.00 ± 87.00 mg/dL, respectively) and returned to control levels within 90 min after SE (117.00 ± 62.93 and 233.00 ± 136.32 mg/dL, respectively). The ovariectomy did not interfere with blood glucose levels when compared to controls groups (OVX and NOVX) (Fig. 2a, Table 1).

**Table 1:** Biochemical and hormonal parameters in *Proechimys guyannensis* submitted to SE induction.

Analytes	CTR		PILO 30		PILO 90	
	NOVX	OVX	NOVX	OVX	NOVX	OVX
Glucose (mg/dL)	124.50 ± 27.34	120.75 ± 11.64	311.25 ± 72.85***	433.40 ± 87.00***	117.40 ± 62.93	233.00 ± 136.32
Cholesterol (Total) (mg/dL)	84.67 ± 12.08	72.00 ± 9.55	68.20 ± 18.20	70.67 ± 24.51	71.67 ± 18.74	57.5 0 ± 12.13
Cholesterol HDL (mg/dL)	42.17 ± 8.45	39.75 ± 8.73	43.40 ± 4.83	46.17 ± 13.41	37.17 ± 11.75	35.50 ± 9.65
Cholesterol not HDL (mg/dL)	42.50 ± 16.53	32.25 ± 18.13	24.80 ± 15.12	24.50 ± 12.98	34.50 ± 24.83	22.00 ± 6.99
Triglycerides (mg/dL)	48.50 ± 10.31	214.50 ± 43.00^###^	59.00 ± 14.25	90.40 ± 21.58***	104.00 ± 35.03**	54.60 ± 16.30***
Sodium (mmol/L)	131.83 ± 13.35	133.75 ± 13.65	142.60 ± 6.69	142.50 ± 5.82	132.33 ± 27.23	127.17 ± 35.98
GGT (U/L)	4.75 ± 1.50	3.25 ± 0.96	3.80 ± 1.48	5.17 ± 2.64	5.20 ± 1.92	6.17 ± 2.48
T3 (free) (ng/dL)	< 40.00 ± 0.00	< 40.00 ± 0.00	44.04 ± 3.41	41.46 ± 4.51	40.13 ± 1.99	42.41 ± 3.92
T4 (total) (µg/dL)	3.68 ± 0.31	3.42 ± 0.41	3.40 ± 0.61	2.82 ± 1.07	3.26 ± 1.28	2.96 ± 0.99
Progesterone (ng/mL)	10.44 ± 4.73	5.84 ± 0.45	22.71 ± 3.12**	11.91 ± 2.71**^##^	10.42 ± 1.53	11.16 ± 5.70**
Oestradiol (pg/mL)	41.00 ± 18.96	28.71 ± 9.46	35.40 ± 9.76	35.00 ± 9.07	32.83 ± 7.78	43.83 ± 11.89
Creatinine (mg/dL)	0.29 ± 0.06	0.18 ± 0.05	0.49 ± 0.04	0.56 ± 0.15	0.52 ± 0.26	0.42 ± 0.32
Urea (mg/dL)	49.75 ± 7.93	54.00 ± 5.65	66.75 ± 3.86	74.50 ± 16.61	66.83 ± 27.01	56.75 ± 25.15

HDL: High Density Lipoprotein; GGT: Gamma-glutamyl transferase; T3: Triiodothyronine; T4: Thyroxine. CTR: Controls animals no submitted to SE; PILO 30: animals analysed 30 min after SE onset; PILO90: animals analysed 90 min after SE onset; NOVX: Non-ovariectomised animals; OVX: Ovariectomised animals. Data are presented as mean ± SD. Two-way ANOVA and Tukey’s post hoc tests were used. **p < 0.01, ***p < 0.001 versus CTR-NOVX if labelled in NOVX group or, versus CTR-OVX if labelled in OVX group. ^##^*p* < 0.01, ^###^*p* < 0.001 versus PILO 30 NOVX and CTR-NOVX, respectively.

Progesterone levels in NOVX animals significantly increased (*p* < 0.001) at 30 min after SE (22.71 ± 3.12 ng/mL) and returned to control levels at 90 min after SE (10.48 ± 1.53 ng/mL). In OVX animals, 30 min after SE onset, the progesterone level significantly increased (11.91 ± 2.71, *p* < 0.05) compared to CTR-OVX (5.84 ± 0.45 ng/mL), and remained increased in 90 min of SE. There was no considerable difference between controls (OVX and NOVX) animals, i.e. the ovariectomy did not lead to a significant decrease in the level of progesterone. When comparing the NOVX with OVX groups, 30 min after SE onset, ovariectomised animals (PILO 30-OVX) showed a significant decrease in progesterone levels (Fig. 2b, Table 1).

In terms of triglyceride levels, there was a significant increase (*p* < 0.001) in the CTR-OVX group (214.50 ± 43.00 mg/dL) compared to the CTR-NOVX group (48.50 ± 10.31 mg/dL). The ovariectomy itself resulted in an increase in triglyceride levels. The higher triglyceride levels could be associated with the lack of sex hormones such as in menopause^19^. After treating OVX animals with pilocarpine, there was a significant decrease in triglycerides in the 30 min (90.40 ± 21.58 mg/dL) and 90 min (54.60 ± 16.30 mg/dL) of SE onset, compared to CTR-OVX (214.50 ± 43.00 mg/dL) (Fig. 2c, Table 1).

The other biochemical and hormonal analyses (cholesterol, gamma-glutamyl transferase [GGT], triiodothyronine [T3], thyroxine [T4], sodium, creatinine, urea, and oestrogen), revealed no statistical differences among the groups (CTR-OVX, PILO 30-OVX, PILO 90-OVX, CTR-NOVX, PILO 30-NOVX, and PILO 90-NOVX; Table 1). These data from female *Proechimys* were similar to the reference values from female Wistars^20,21^.

### Ovariectomy produced changes in the hippocampus of *Proechimys* with characteristics of vulnerability to seizures

**Figure 3:**
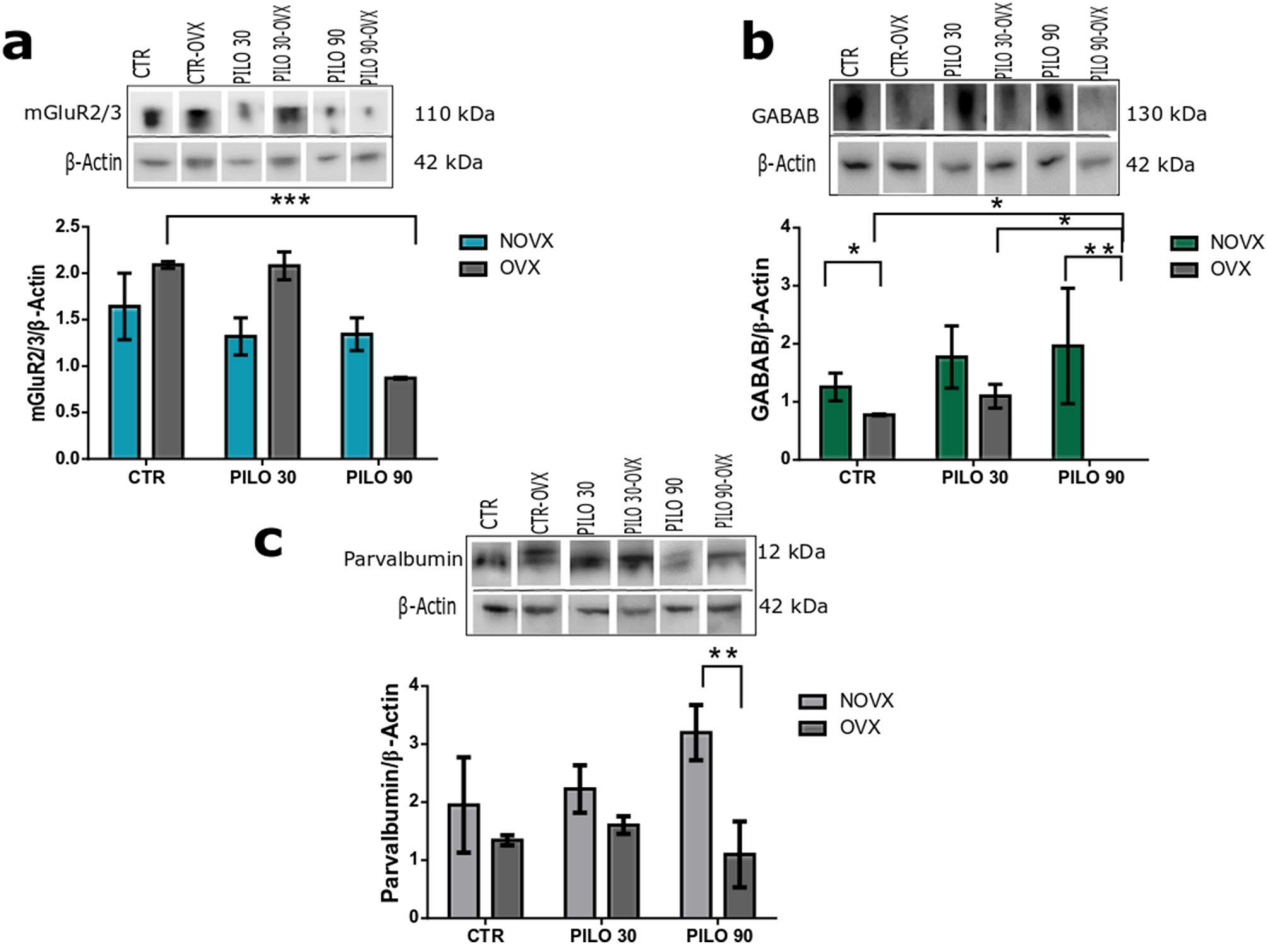
Analysis of hippocampal GABAergic and glutamatergic receptor expression in NOVX and OVX animals following pilocarpine treatment. GABAergic and glutamatergic receptor expression was decreased in OVX animals treated with pilocarpine 90 min after status epilepticus (SE) onset. No changes were noted in parvalbumin receptor expression that could be linked to SE. (**a**) Metabotropic glutamate receptor 2/3 (mGluR2/3) expression. (**b**) GABA_B_ receptor expression. (**c**) Parvalbumin receptor expression. CTR: Control; NOVX: Non-ovariectomised animals; OVX: Ovariectomised animals. β-Actin was used for normalisation. All hippocampal samples were run through the gel simultaneously (uncropped gels are presented in Supplementary Information B as Figures S1–S3). Data are presented as mean ± SD. Levels marked with asterisk (*) indicate significant differences: *p < 0.05, **p < 0.01, ***p < 0.001.

The expression of the mGluR2/3 excitatory receptor in the hippocampus showed no change in NOVX animal after pilocarpine treatment. In the OVX animals, the receptor significant decrease (*p* < 0.001) in the PILO 90-OVX group as compared to the CTR-OVX group. When comparing CTR-NOVX and CTR-OVX groups, although there was no difference between groups, there was a tendency for glutamate receptors to be increased in OVX animals (Fig. 3a).

The analysis of GABA_B_ inhibitory receptor expression in the hippocampus showed no change in NOVX groups (CTR, PILO 30, and PILO 90) after pilocarpine treatment. However, the CTR-OVX group (0.77 ± 0.02) showed a significant decrease in GABA_B_ levels in the PILO 90-OVX group (the expression of GABA_B_ receptors was reduced below the detection level) (Fig. 3b). When comparing CTR-NOVX and CTR-OVX groups, the CTR-OVX group showed a significant decrease in GABA_B_ levels (*p* < 0.05).

The expression of parvalbumin receptors was also analysed in the hippocampus due to its critical role in generating network oscillations and its involvement in epileptic seizures. There was no difference among the groups, except for the PILO 90-OVX group, compared to PILO 90-NOVX, which showed a decreased (*p* < 0.01) expression of these receptors. This may demonstrate a weakness in this type of GABAergic neuron at 90 min after SE onset, in ovariectomised *Proechimys*. This emphasises that no differences were found between CTR-NOVX and CTR-OVX groups (Fig. 3c).

### Changes in progesterone and oestrogen receptor patterns in the hippocampus

**Figure 4:**
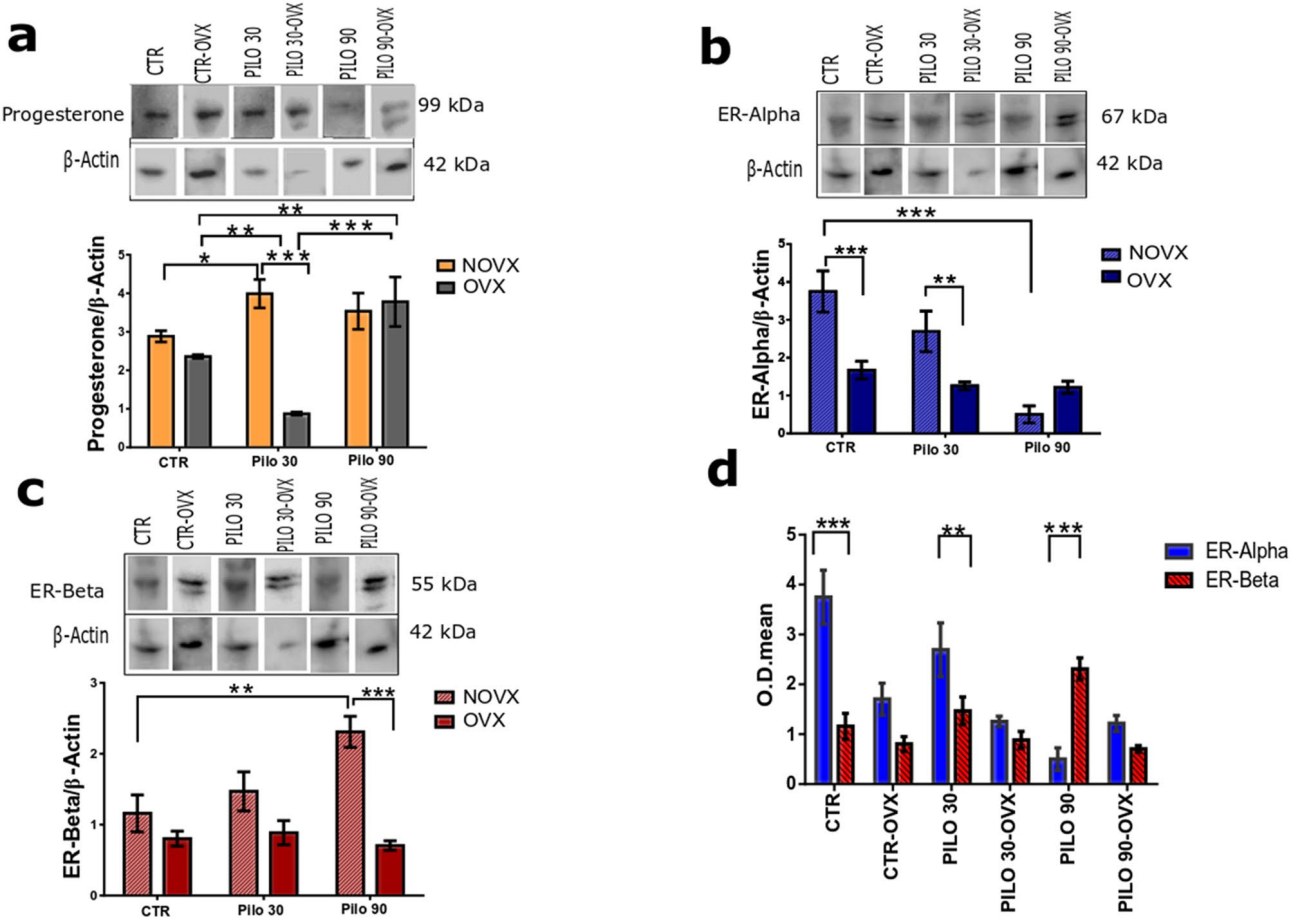
Changes in hippocampal progesterone and oestrogen receptor expression in NOVX and OVX animals following pilocarpine treatment. Progesterone receptor expression increased along the status epilepticus (SE) timeline (30 min for NOVX and 90 min for OVX). Within 90 min of SE onset, the expression of oestrogen receptor (ER)-alpha decreased and that of ER-beta increased markedly in NOVX animals. As such, ER-alpha expression was higher than ER-beta expression in both OVX and NOVX animals. (**a**) Progesterone receptor expression. (**b**) ER-alpha expression. (**c**) ER-beta expression. (**d**) Comparative optical densities (OD) between ER-alpha and ER-beta. CTR: Control; NOVX: Non-ovariectomised animals; OVX: Ovariectomised animals. β-Actin was used for normalisation. All hippocampal samples were run through the gel simultaneously (uncropped gels are presented in Supplementary Information B as Figures S4–S6). Data are presented as mean ± SD. Levels marked with asterisk (*) indicate significant differences: *p < 0.05, **p < 0.01, ***p < 0.001.

The analysis of progesterone receptor patterns revealed differences between the PILO 30 and CTR among NOVX groups, with an increased at 30 min (*p* < 0.05). In contrast, there was a reduction in the progesterone level expression in the PILO 30-OVX group (*p* < 0.01) and an increase expression in the PILO 90-OVX group (*p* < 0.01), as compared to the CTR-OVX group (Fig. 4a).

Regarding oestrogen receptor patterns during SE, in the NOVX groups, the ER-alpha expression was significantly decreased (*p* < 0.001) at 90 min after SE onset, compared to CTR-NOVX. In OVX animals, no statistical differences were found among CTR-OVX, PILO 30-OVX, and PILO 90-OVX groups (Fig. 4b). Ovariectomy by itself decreases ER-alpha expression in the hippocampus, according to comparative data between CTR-NOVX and CTR-OVX groups. An ovariectomy by itself did not interfere with the expression of ER-alpha in the hippocampus.

With regards to the ER-beta, the expression in the NOVX group significantly increased (*p* < 0.01) at 90 min after SE onset, compared to the CTR-NOVX group. In OVX animals, no statistical differences were found among CTR-OVX, PILO 30-OVX, and PILO 90-OVX groups (Fig. 4c). When analysing the proportion of ER-alpha receptors in relation to ER-beta expression in the hippocampus, the expression of ER-alpha was greater than that of ER-beta, except for PILO 90-NOVX group, which had a higher expression of ER beta than ER-alpha (Fig. 4d).

### Rearrangement of receptors in the *Proechimys* hippocampus after pilocarpine

**Figure 5:**
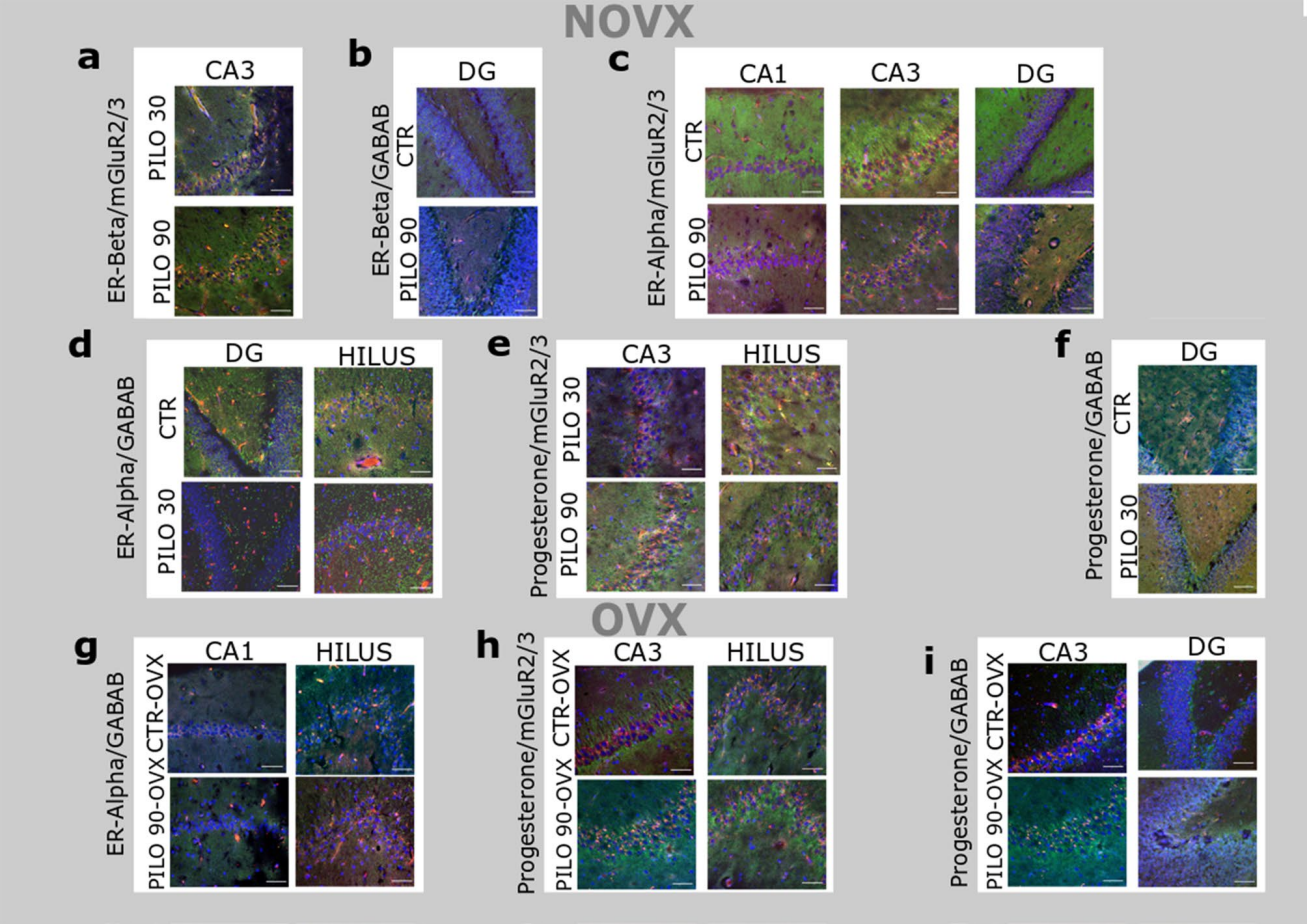
Photomicrographs of hippocampal regions (CA1, CA3, DG, and hilus) of *Proechimys guyannensis* with significantly different receptor co-localisations. Sections processed for(**a**) ER-beta (red) and mGluR2/3 (green) immunofluorescence (**b**) ER-beta (red) and GABA_B_ (green) immunofluorescence; (**c**) ER-alpha (red) and mGluR2/3 (green) immunofluorescence; (**d**) ER-alpha (red) and GABA_B_ (green) immunofluorescenc; (**e**) progesterone (red) and mGluR2/3 (green) immunofluorescence; (**f**) progesterone (red) and GABA_B_ (green) immunofluorescence in NOVX *Proechimys.* Sections processed for (**g**) ER-alpha (red) and GABA_B_ (green) immunofluorescence; (**h**) progesterone (red) and mGluR2/3 (green) immunofluorescence; (**i**) and progesterone (red) and GABA_B_ (green) immunofluorescence in OVX *Proechimys*. CTR: Control; NOVX: Non-ovariectomised animals. OVX: Ovariectomised animals. Scale bars = 100 μm.

We also co-stained *Proechimys* hippocampi for the hormonal ER-alpha, ER-beta, and progesterone receptors (stained in red) with GABA_B_ and mGluR2/3 receptors (stained in green; Fig. 5a–i). The co-expression of ER-alpha, ER-beta, and progesterone receptors with GABA_B_ was detected in all analysed hippocampal regions. Likewise, the co-expression of ER-alpha, ER-beta, and progesterone with mGluR2/3 was analysed; progesterone receptor co-expression with mGluR2/3 was only observed in the cell membrane, whereas ER-alpha and ER-beta co-expression was detected in the nucleus and the cell membrane. The co-localisations patterns were quantified in the hippocampal areas: CA1, CA3, DG, and hilus (Table 2). Figure 5 outlines the statistical differences found at 30 min and 90 min after SE onset.

**Table 2:** Colocalization of oestrogen and progesterone receptors with GABA_B_ and mGluR2/3 receptors in the hippocampi of females *Proechimys guyannensis.*

Groups	GABA_B_				mGluR2/3			
	CA1	CA3	DG	Hilus	CA1	CA3	DG	Hilus
**ER-alpha**								
CTR-NOVX	0.77 ± 0.005	0.81 ± 0.01	0.72 ± 0.01	0.73 ± 0.005	0.34 ± 0.01	0.54 ± 0.01	0.46 ± 0.03	0.64 ± 0.006
CTR-OVX	0.87 ± 0.005	0.76 ± 0.03	0.80 ± 0.006	0.60 ± 0.006	0.59 ± 0.006	0.55 ± 0.01	0.68 ± 0.01	0.58 ± 0.01
PILO 30-NOVX	0.78 ± 0.01	0.78 ± 0.01	0.46 ± 0.02**	0.47 ± 0.005**	0.58 ± 0.06	0.60 ± 0.006	0.55 ± 0.07	0.64 ± 0.006
PILO 30-OVX	0.75 ± 0.004	0.81 ± 0.006	0.72 ± 0.01	0.73 ± 0.01	0.63 ± 0.01	0.53 ± 0.06	0.53 ± 0.05**	0.48 ± 0.08
PILO 90-NOVX	0.76 ± 0.01	0.82 ± 0.03	0.68 ± 0.01	0.72 ± 0.005	0.66 ± 0.01**	0.65 ± 0.006**	0.71 ± 0.006**	0.60 ± 0.006
PILO 90-OVX	0.71 ± 0.03*	0.74 ± 0.01	0.66 ± 0.01**	0.78 ± 0.03**	0.54 ± 0.01^##^	0.50 ± 0.006	0.62 ± 0.006	0.48 ± 0.02
**ER-beta**								
CTR-NOVX	0.05 ± 0.005	0.18 ± 0.01	0.05 ± 0.006	0.04 ± 0.006	0.23 ± 0.08	0.22 ± 0.01	0.35 ± 0.06	0.22 ± 0.01
CTR-OVX	0.40 ± 0.002	0.43 ± 0.01	0.46 ± 0.006	0.43 ± 0.006	0.21 ± 0.01	0.39 ± 0.05	0.36 ± 0.03	0.23 ± 0.01
PILO 30-NOVX	0.27 ± 0.01**	0.35 ± 0.006**	0.19 ± 0.01	0.28 ± 0.05*	0.07 ± 0.01*	0.21 ± 0.01	0.24 ± 0.02	0.24 ± 0.01
PILO 30-OVX	0.20 ± 0.006*	0.21 ± 0.006*	0.38 ± 0.01	0.26 ± 0.03	0.19 ± 0.01	0.26 ± 0.02**	0.24 ± 0.02	0.24 ± 0.01
PILO 90-NOVX	0.17 ± 0.02	0.29 ± 0.03	0.29 ± 0.01**	0.25 ± 0.06	0.15 ± 0.01	0.29 ± 0.01^#^	0.24 ± 0.01	0.23 ± 0.01
PILO 90-OVX	0.21 ± 0.01*	0.25 ± 0.006	0.26 ± 0.01**	0.19 ± 0.01**	0.19 ± 0.01	0.33 ± 0.01	0.24 ± 0.00	0.20 ± 0.01
**Progesterone**								
CTR-NOVX	0.37 ± 0.006	0.51 ± 0.07	0.35 ± 0.01	0.40 ± 0.01	0.20 ± 0.003	0.38 ± 0.02	0.44 ± 0.01	0.24 ± 0.01
CTR-OVX	0.46 ± 0.01	0.55 ± 0.02	0.57 ± 0.04	0.90 ± 0.006	0.36 ± 0.1	0.54 ± 0.005	0.50 ± 0.07	0.58 ± 0.01
PILO 30-NOVX	0.37 ± 0.006	0.52 ± 0.01	0.05 ± 0.006**	0.48 ± 0.09	0.29 ± 0.01	0.63 ± 0.09	0.36 ± 0.006	0.67 ± 0.01*
PILO 30-OVX	0.42 ± 0.01	0.46 ± 0.01	0.09 ± 0.005	0.81 ± 0.01**	0.75 ± 0.02	0.48 ± 0.03	0.76 ± 0.01	0.57 ± 0.006
PILO 90-NOVX	0.27 ± 0.02	0.43 ± 0.005	0.06 ± 0.006	0.46 ± 0.005	0.35 ± 0.01**	0.34 ± 0.01^##^	0.40 ± 0.02	0.73 ± 0.006**
PILO 90-OVX	0.40 ± 0.02	0.42 ± 0.01**	0.08 ± 0.005*	0.89 ± 0.006	0.24 ± 0.01^##^	0.39 ± 0.02**	0.30 ± 0.03^#^	0.44 ± 0.01*

ER-alpha: Oestrogen receptor alpha; ER-beta: Oestrogen receptor beta; GABA_B_: Gamma-aminobutyric acid; mGluR2/3: Metabotropic glutamate receptor; CTR-NOVX: Non-ovariectomised control group, animals no submitted to SE; CTR-OVX: Ovariectomised control group, animals no submitted to SE; PILO 30-NOVX, Non-ovariectomised animals analysed 30 min after SE onset; PILO 30-OVX: Ovariectomised animals analysed 30 min after SE onset; PILO 90-NOVX, Non-ovariectomised animals analysed 90 min after SE onset; PILO 90-OVX: Ovariectomised animals analysed 90 min after SE onset. Data are presented as mean ± SD. Kruskal–Wallis test followed by Dunn’s post hoc test. *p < 0.05, **p < 0.01 versus CTR-NOVX or CTR-OVX. ^#^*p* < 0.05, ^##^*p* < 0.01 versus PILO 30-NOVX or PILO 30-OVX.

When comparing the co-localisations patterns of receptors in PILO 30 (NOVX or OVX) and PILO 90 (NOVX or OVX) with the respective control groups NOVX or OVX, we observed several receptor reorganization trends.

In NOVX animals, ER-beta/mGluR2/3 co-localisation was low in the CA1 region of PILO 30-NOVX (0.07 ± 0.01) rodents compared with CTR-NOVX (0.23 ± 0.08) animals. ER-beta/GABA_B_ co-localisation was elevated in the CA1 (0.27 ± 0.01), CA3 (0.35 ± 0.006) and hilus (0.28 ± 0.05), compared to respective CTR-NOVX (Table 2). Additionally, ER-beta/GABA_B_ co-localisation was elevated in the DG (0.29 ± 0.01) of the PILO 90-NOVX group compared with the CTR-NOVX group.

ER-alpha/mGluR2/3 co-localisation in the PILO 90-NOVX group was increased in the CA1 (0.66 ± 0.01), CA3 (0.65 ± 0.01), and DG (0.71 ± 0.01) as compared with the respective CTR-NOVX group (Table 2), whereas ER-alpha/GABA_B_ co-localisation was reduced in the DG (0.46 ± 0.02) and hilus (0.47 ± 0.01) in PILO 30-NOVX as compared with CTR-NOVX animals.

The co-localisation of progesterone/mGluR2/3 was higher in the hilus (0.67 ± 0.01) regions of PILO 30-NOVX and the higher in CA1 (0.35 ± 0.01) and hilus (0.73 ± 0.006) regions of PILO 90-NOVX, as compared to the respective control group. The progesterone/GABA_B_ co-localisation was decreased in the DG (0.06 ± 0.06) of the PILO 30-NOVX group as compared with the CTR-NOVX group (Table 2).

**Figure 6:**
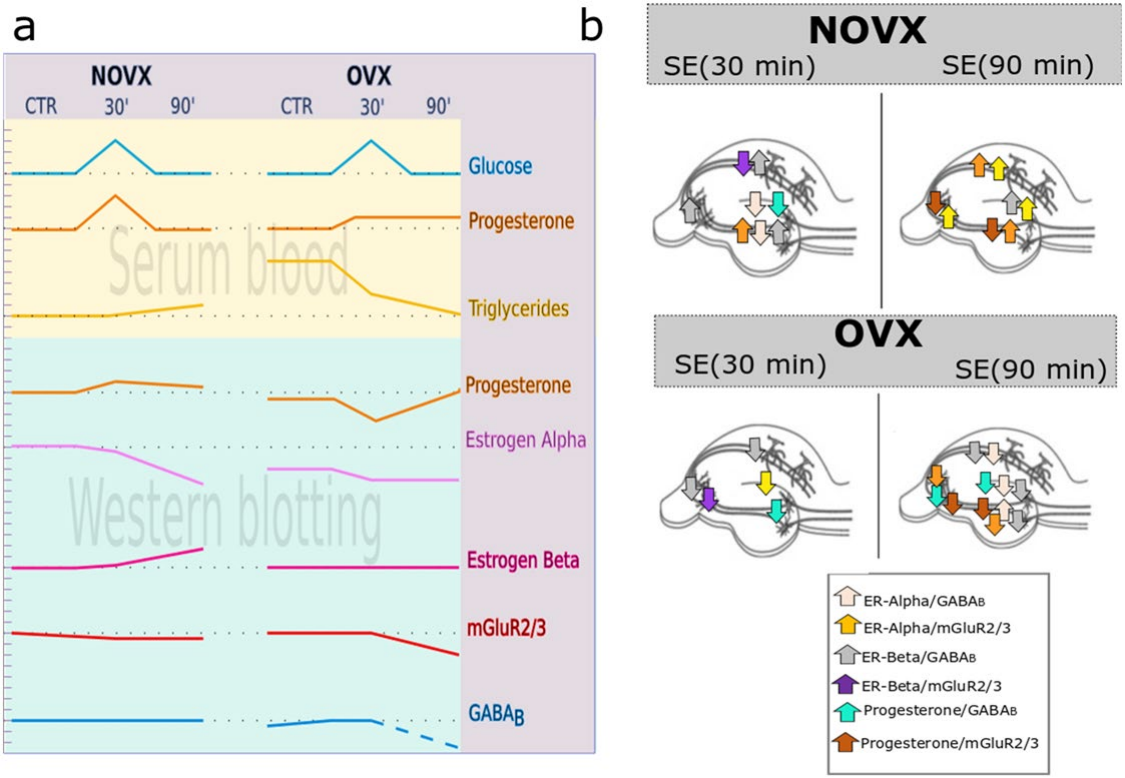
Simplified scheme of biochemical changes in blood serum, western blots, and photomicrographs of hippocampi of *Proechimys guyannensis* female with or without ovariectomy. (**a**) Biochemical and hormonal changes in serum and protein expression in NOVX and OVX animals with status epilepticus (SE). (**b**) Co-localisation differences in the hippocampus of *P. guyannensis* between control groups (NOVX and OVX)*.* NOVX: Non-ovariectomised animals; OVX: Ovariectomised animals; CTR: Control, animals that have not been subjected to SE; 30′: animals analysed 30 min after SE onset; 90′: animals analysed 90 min after SE onset.

In OVX rodents, all changes can be seen in Table 2 and Fig. 6.

## Discussion

To the best of our knowledge, this study is a pioneer in using females *P. guyannensis* for SE induction analyses. Comparative analyses were performed with NOVX and OVX *Proechimys* undergoing on SE, and respective controls (CTR-NOVX, CTR-OVX) were analysed. Female Wistars were used as a positive control.

There is a strong incentive from the National Institutes of Health to include females because results will be more consistent with the general population^15^. We conducted studies in female *Proechimys* (NOVX group) selecting the oestrus stage to minimise the bias related to hormonal changes. In our previous work analysing the oestrous cycle of *Proechimys*, low levels of progesterone were observed in the oestrus stage^14^, which can correspond to the progesterone withdrawal phenomenon leading to the increased excitability and susceptibility to seizures, as proposed to explain the increase in seizures that usually occur during the perimenstrual period in women with epilepsy^22,23^. In this context, the oestrus stage chosen to carry out this work would be more closely associated with a decrease than an increase in the threshold for SE induction.

As result of pilocarpine treatment, the mean SE latency in NOVX was no longer than that in female Wistar rats, but the dose of pilocarpine required to reach SE was higher for *Proechimys* (25- to 50-folds more) (Fig. 1). During SE, *Proechimys* evolved only up to stage 2 on the Racine scale, unlike Wistar rats which usually reach stage 5 of this scale—the lower the scale, the less strong SE. The pilocarpine dose to induce SE, latency to SE and the Racine scale were similar to NOVX and OVX *Proechimys*. Ovariectomised animals remain resistant to induced seizures, i.e. ovarian hormones do not appear to have interfered with seizure threshold and intensity in *Proechimys.* This result is in contrast to our previous study in Wistar rats, in which ovariectomy decreases the latency to SE, indicating that these rodents without ovaries became more sensitised to pilocarpine^9^.

The systemic injection of pilocarpine in rodents is one of the most frequently used models for evoking experimental SE^24,25^. Pilocarpine-induced SE results from acute peripheral proinflammatory actions of pilocarpine leading to blood–brain barrier leakage, as well as a direct cholinergic system activation by pilocarpine in the brain^26^. The dose of pilocarpine influences substantially the induced effects. The pilocarpine dose schedule is critical for obtaining animals experiencing SE with minimal mortality. We perform the lithium-pilocarpine ramp-up protocol due to its high proportion of rodents developing epilepsy and its low mortality (Supplementary Information A). In female Wistar, pretreatment with lithium results in a conspicuous reduction in the pilocarpine dose required to induce SE: 320 mg/kg without lithium^9^ and 10 mg/kg with lithium (present study).

In female *Proechimys*, pretreatment with lithium results in a higher dose required to induce SE (250 to 500 mg/kg) compared to those used in female Wistar rats. Surprisingly, lithium may not potentiate the convulsive effects of pilocarpine in female *Proechimys*, similar to observations obtained in mice^27,28^. Although an explanation for the lack of lithium potentiating the susceptibility of mice to pilocarpine has not been provided, it may be due to species differences in the modulation of inflammatory activity, glutamate receptors^29^, or the lack of inositol depletion by lithium^30,31^.

Previous studies in male *Proechimys*, epileptogenic treatment with pilocarpine without lithium, hippocampal inflammatory cytokines (IL-1β, IL-6, IL-10, and TNF-α) and vascular endothelial growth factor remain unchanged after SE^32^, suggesting that *Proechimys* exhibit attenuated inflammatory responses to injury. Furthermore, although male *Proechimys* develop SE, they do not become epileptic in contrast to Wistar rats. In these animals, the SE rarely exceeds 2 h, in contrast to the 8–12 h in Wistar rats^13^.

The SE undergoes temporal stages based on pathophysiological changes and the relationship between SE duration and epilepsy development. Hippocampal damage with subsequent complex synaptic reorganization is only produced by more than 30 min of SE^33^. Temporal changes occur in the brain during the progression of tonic–clonic SE; around 30 min after SE onset, physiological changes are compensatory. With prolonged SE, these compensatory mechanisms break down. As the SE continues for more than 60 min, the risk of brain damage progressively increases along with the depletion of glucose and glycogen concentrations in the brain^34^.

Here, we assess serum blood and hippocampus at 30 and 90 min after SE onset, to identify changes that may contribute to SE resistance. Anti-epileptogenesis in *Proechimys*. *Proechimys* (NOVX and OVX) presented high glucose levels at 30 min of seizures, and at 90 min of seizures glucose levels remained equal to controls (CTR-NOVX and CTR-OVX, respectively) (Figs. 2a, 6a). This differs from other rodents and humans presenting a decrease in blood glucose after 30 min of SE^35,36^.

It has already been suggested that glucose regulation may be a critical factor linked to the susceptibility to seizure-induced cell death^36^. Glucose metabolism is impaired in humans under SE^37^, and some seizures usually improve with glycaemic control^38,39^. Cholinergic agents, as pilocarpine, are known to activate insulin production in rodents^40,41^ which leads to hypoglycaemia. In *Proechimys*, treatment with pilocarpine generated hyperglycaemia rather than hypoglycaemia. In hormonal analyses, the equipment was unable to measure the insulin concentration in the blood serum of *Proechimys*. The reason underlying this phenomenon remains unknown, and may be linked to the lack of immunospecificity of the antibody to detect *Proechimys* insulin.

The ability of *Proechimys* to maintain glucose homeostasis along with the apparent recovery from seizures after SE induction is peculiar (pilot study findings). A different scenario involves the homeostasis of glucose in *Proechimys*. Atypically, the glucose regulation mechanism in these animals is not based on the action of insulin, as it normally occurs in mammals. *Proechimys* have an insulin molecule with amino acid substitutions that confers a lower potency (< 10%) compared to bovine insulin^42–44^. Consequently, this leads to an impaired binding affinity for insulin receptors in the hepatic plasma membrane and an inability to stimulate glucose uptake and, thus, its function in lipid conversion^43^.

However, *Proechimys* regulate glucose levels like other mammals^45^, suggesting compensatory traits that may permit maintenance of standard values of plasma glucose^45^. May *Proechimys* resistance to epilepsy be caused by an unknown mechanism that regulates their glucose? Glucose homeostasis is performed by a highly sophisticated network of hormones and neuropeptides released mainly from the pancreas, brain, liver and intestine^46,47^. Molecules, enzymes and interactions may triggers signalling pathways underlying insulin secretion^47^.

Analysing the biochemical and hormonal blood concentrations, NOVX and OVX *Proechimys* behaved similarly. The only difference found in the blood serum between CTR-OVX and CTR-NOVX was the increase in the level of triglycerides in the OVX animals. Interestingly, even without the ovaries, ovarian hormones are present in OVX animals. Moreover, OVX increases progesterone levels during SE, as NOVX. Progesterone is an anticonvulsant hormone^48^ that can be synthesised by the adrenal glands and the nervous system, besides being synthesised in the ovary^46^. Usually, in *Proechimys*, the highest level of progesterone is found in the proestrus stage, lasting 2 to 3 days^14^. The increase in progesterone in the blood serum over a short time period, over the course of 30 min of SE, was an interesting finding, seen mainly in the OVX animals (Fig. 2b).

Regarding the hippocampal analysis, an ovariectomy by itself leads to a decrease in GABA_B_ receptors. In the hippocampus, neuronal network is composed of excitatory (glutamatergic) and inhibitory (GABAergic) neurons, the imbalance in this network leads to seizures. In *Proechimys* NOXV treated with pilocarpine, expressions of glutamatergic (mGluR2/3) or GABAergic (GABA_B_) receptor did not change. When ovariectomized animals received pilocarpine, a decrease in mGluR2/3 and GABA_B_ occurred at 90 min of SE. So far, it is possible that the ovariectomy in these animals leads to the vulnerability of GABAergic and glutamatergic neurons if the SE lasts 90 min (Fig. 3a,b).

GABA_B_ receptors present in inhibitory neurons are modulators of the short-term plasticity of excitatory synapses^49^. Several types of inhibitory neurons (interneurons) are found in the hippocampus with peculiar properties regarding neuronal discharge patterns. Positive parvalbumin interneurons are involved in the generation of network oscillations and may play a key role in seizures^50^. We analysed parvalbumin receptor expression in the OVX *Proechimys* hippocampus to assess whether any remodelling in the interneuronal network could be occurring during SE. In this study, SE did not involve the remodelling of parvalbumin interneurons.

When analysing hormonal receptors in the hippocampus, changes in the expression patterns of progesterone and oestrogen receptors were found in NOVX and OVX *Proechimys*, according to the treatment. After SE induction, expression of progesterone receptor increased in 30 min for NOVX and 90 min for OVX (Fig. 4a). This increase in 90 min in OVX may be related to the local synthesis of progesterone by glial and neuronal cells^51,52^. Which may indicate optimised use of progesterone by the hippocampus. Progesterone is an anticonvulsant agent with neuroprotective role, *Proechimys* may also use this mechanism in an attempt to protect themselves from seizures.

We also analysed the expression patterns of oestrogen receptor in the hippocampus, although there was no increase in the serological level during SE, the increase in expression of oestrogen receptor can make the hormone more bioavailable. A higher ER-alpha than ER-beta expression was found (CTR-NOVX; Fig. 4d), as in mice^53^. On the other hand, ER-beta is more prevalent in humans and rats hippocampi^54–56^. Through these receptors, oestrogen activates rapid signalling in neurons^57^ and both oestrogen receptors (ER-alpha and -beta) seem to be involved in neuroprotection^58–60^. ER-alpha is known to plays a critical role in regulating reproductive neuroendocrine function^61^. ER-beta agonist significantly reduce damage by 55% in the CA1 region of hippocampus after injury^60^, this receptor is crucially involved in hormonal and behavioural responses associated with stress^62,63^. A decrease in ER-alpha and an increase in ER-beta occurred within 90 min of SE onset; in NOVX animals (Fig. 4b,c, respectively), the pilocarpine treatment and the following seizures may be the causes of this receptor reorganization.

Although ovariectomy in *Proechimys* does not influence the SE, changes in protein receptor expression patterns are observed in the brain. Analysing different areas of the hippocampal formation (CA1, CA3, DG ,and hilus) using immunohistochemistry, differences were found between the OVX and NOVX groups showing that ovarian hormones may play an important role in hippocampal rearrangements at SE induction (Fig. 5, Table 2). We made schematic resume of all the changes found in this study relation to serum, western blot and immunohistochemistry (Fig. 6a,b).

Finally, although the latency to SE observed in female *Proechimys* is similar to that in other rodents, the dose of pilocarpine required to induce SE was higher in *Proechimys* than in Wistar rats. Moreover, the intensity of seizures in *Proechimys* is lower when compared to other rodents, measured on the Racine scale. Of all biochemical analyses, glucose and progesterone appear to have an important influence. We can speculate whether the mechanism behind the glucose compensation during SE may be a part of the compensatory mechanisms that make the induction of seizures in these rodents difficult. Rearrangements that occur in the hippocampus (in mGluR2/3, GABA_B_, progesterone, ER-alpha and ER-beta receptors) may slightly interfere with SE. A decrease in GABAergic receptors within 90 min of SE in OVX animals may suggest ovariectomy produces changes in the hippocampus with characteristics of vulnerability to seizures. SE episodes commonly lead to recurrent seizures and epilepsy. This rodent, with an unknown mechanism underlying the innate resistance to SE, thereby represents a special opportunity to better understand SE.

## Methods

*Proechimy*s rodents were bred in a colony established at Universidade Federal de São Paulo (UNIFESP). All animals handling and experimental procedures complied with the guidelines for animal care and use of laboratory animal and were approved by the Ministry of the Environment (IBAMA—Instituto Brasileiro do Meio Ambiente e dos Recursos Naturais Renováveis), protocol nº 1561643, and by the Board for Ethics in the Use of Animals (CEUA, Comissão de Ética no Uso de Animais), an institutional ethics committee of the UNIFESP, protocol nº 5594280316.

### Animals

Thirty-six female virgin *P. guyannensis* rodents (age, 1 year; weight, 210–280 g), were housed under controlled conditions at 21 ± 2 °C in a light/dark cycle of 12/12 h with food and water ad libitum. *Proechimys* rodents were randomly allocated to six groups of six animals each (Fig. 2d). Three groups contained non-ovariectomised (NOVX) rodents in the oestrus stage (CTR, treated with lithium and saline; PILO 30, lithium-pilocarpine-induced SE for 30 min; and PILO 90, lithium-pilocarpine-induced SE for 90 min), whereas the other three groups consisted of ovariectomised (OVX) rodents with corresponding SE induction (CTR-OVX, PILO 30-OVX, and PILO 90-OVX, respectively).

Based on our published study on characterization of the oestrous cycle in female *Proechimys*^14^, oestrus stage was chosen in the current work to carry out analyses on NOVX *Proechimys* due to low levels of progesterone observed.

### Ovariectomy

Bilateral flank incisions were performed in 18 rodents anaesthetised with 10% ketamine hydrochloride (150 mg/kg, ip) and 2% xylazine hydrochloride (11 mg/kg, ip). The ovaries were removed, and the uterine horns were replaced. The muscular wall and skin were closed using absorbable and non-absorbable sutures, respectively. Rodents were treated with tramadol hydrochloride (5 mg/kg, ip) as an analgesic and enrofloxacin (5 mg/kg, im) as an antibiotic for 3 days. The ovariectomy success was verified 1 week after surgery through vaginal smears. Ovariectomies were performed 1 month before SE induction.

### SE induction

*Proechimys* were treated with lithium chloride (127 mg/kg, ip) and methylscopolamine-bromide (1 mg/kg, ip, both Sigma-Aldrich) 24 h and 30 min, respectively, before pilocarpine injections. The ramp-up pilocarpine protocol^17^ was adapted for *Proechimys* rodents; pilocarpine (250 mg/kg, ip) was administrated every 30 min until SE developed. This model was chosen as it efficiently induces SE with low mortality rates^17^. Seizures were scored using Racine's scale^18^ . The beginning of the SE was defined as the onset of continuous generalised seizure activity without regaining normal behaviour, and pilocarpine injections were limited to five injections per animal. The SE was terminated by euthanasia of the animals after 30 or 90 min, and the brains were quickly removed.

### Serum analysis

The animals were decapitated without anaesthesia for the blood collection. After 30 min, the blood samples (n = 36) were centrifuged (239×*g*, 15 min, 24 °C), and the serum was stored at – 80 °C until analysed. The measurement of glucose, triglycerides, total cholesterol, and fractions (HDL, not HDL), GGT, T3, T4, sodium, creatinine, urea, progesterone, and oestradiol was outsourced to a clinical laboratory. The biochemistry parameters were analysed with ADVIA-2400 (Siemens, NY) equipment to determine glucose, triglycerides, total cholesterol, HDL, not HDL, GGT, and creatinine. The urease enzyme method was used to quantify urea, and the ion–electron selective method was used for sodium determination (Aptec-Biosys Ltda, Belgium). The chemiluminescence method (Beckman Coulter, Unicel DXI-800, USA) was performed for T3, T4, oestradiol, and progesterone.

### Western blots

The hippocampi (n = 3 per group) were dissected and stored at –80 °C until assayed. Samples were homogenised in lysis buffer (5 M NaCl, 0.5 M HEPES, 0.5 M EDTA, pH 8.0, 1% Nonidet P-40) with protease inhibitor cocktails (Sigma-Aldrich). The protein content was determined using the Bradford method^64^. Samples were diluted in Laemmli buffer and boiled for 5 min. Six standard curves were generated using several protein concentrations. Protein samples of 40 μg for ER-alpha, ER-beta, mGluR2/3, 15 μg for progesterone and GABA_B_ receptor, and 60 μg for parvalbumin were within the linear range of this assay. Thus, an equivalent amount of protein was electrophoresed on 10% polyacrylamide mini-gels (8–16% gel for parvalbumin) and transferred to 0.45 µm nitrocellulose membrane sheets (Millipore) through electroblotting. Samples (NOVX and OVX) were load in two gels, it was unable to load all sample in one single gel. The gels were run in parallels. The procedure and loading of all the samples were carried out in a similar fashion. To avoid any mistakes, we load one sample of control NOVX on each gel and compare all band intensities to the intensity of the standard. Finally, all bands were normalised with housekeeping protein (β-Actin) (uncut gels can be found in Supplementary Information B).

Membranes were blocked with 5% non-fat milk (15 min, 24 °C) and incubated with mouse monoclonal antibodies against ER-alpha (1:500; Novus, NR3A1), ER-beta (1:2500; Novus, NR3A2), progesterone (1:1000; Abcam, ab2765), or parvalbumin (1:1000; Sigma, P3088) or rabbit polyclonal against mGluR2/3 (1:1,000; Novus, NB100-1760) or GABA (1:500; Sigma, A2052) in TBS‐T (50 mM Tris‐HCl, 154 mM NaCl, pH7.5, 0.1% Tween 20) plus 2% non-fat milk at 4 °C overnight. Afterwards, blots were washed with TBS-T and incubated for 1 h with peroxidase-conjugated anti-mouse or anti-rabbit immunoglobulin (Calbiochem), diluted in TBS‐T plus 2% non-fat milk.

Blots were washed in TBS-T and enhanced chemiluminescence reagents (Amersham Pharmacia Biotech) were applied to the blots. The membranes were then exposed to the chemiluminescence imaging system (UVITEC), and the bands were quantified by densitometry. Anti-β-actin immunoglobulins (1:30,000; Sigma) were used as controls. The ratios between the optical densities of the NOVX or OVX groups for each receptor and the internal standard of β-Actin bands are presented as mean ± SD.

### Staining preparations

*Proechimys* brains (n = 3 per group) were removed immediately and fixed in 0.1 M phosphate-saline buffer solution (PBS; pH 7.4) containing 4% formaldehyde for 24 h at 4 °C. The brains were soaked in a cryoprotectant solution containing 30% sucrose in PBS, then cut in 40-μm coronal sections using a freezing microtome. Sections were stored individually with an anti-freezing solution (30% sucrose, 1% polyvinylpyrrolidone 40, and 30% ethylene glycol in PBS, pH 7.2). Every sixth coronal slice was kept in sequential order in that anti-freezing solution until used.

For immunofluorescence, adjacent sections were selected. Three hippocampal sections per animal were mounted on slides for further dehydration and dyeing. Images were taken using an OLYMPUS BX60 microscope with a 20× objective and the SPOT software, version 4.6 (http://www.spotimaging.com). The classical subdivisions of the hippocampus (CA1, CA3, dentate gyrus (DG), and hilus) were identified using stereotactic coordinates^65^ .

### Immunofluorescence

A double-labelling protocol was used to examine the co-expression of ER-alpha, ER-beta, or progesterone receptors with GABA_B_ or glutamate (mGluR2/3) receptors. Selected brain slices were washed with 0.1 M PBS (pH 7.4), then placed in a blocking solution (containing 10% foetal bovine serum, 2% albumin, and 0.1% Triton-X) for 120 min. Afterwards, the slices were incubated with primary mouse monoclonal antibodies against ER-alpha (1:500), ER-beta (1:500), or progesterone (1:50) for 48 h at 4 °C. The primary antibodies were removed by several washes in 0.1 M PBS. Then, the slices were incubated with Alexa Fluor 594-coupled secondary antibodies (red, Invitrogen) for 30 min.

Next, the slices were washed again with 0.1 M PBS and incubated with the second primary rabbit polyclonal anti-GABA_B_ antibody (1:5000) or mGluR2/3 (1:500) overnight at 4 °C. The next day, the slices were washed again with 0.1 M PBS and incubated with the Alexa Fluor 488-conjugated secondary antibody (green, Invitrogen). Sections were washed in PBS, coverslipped with FluoroShield mounting medium containing DAPI (Sigma, F6057), and stored at 4 °C to preserve the fluorescence. Samples were analysed using the confocal microscope TCS SP8 (Leica) with a 40× objective. Images were imported into Fiji (http://fiji.sc, a version of the ImageJ software, National Institutes of Health, http://imagej.nih.gov/ij). The coloc2 plugin was used to analyse the colocalization of ER-alpha, ER-beta, or progesterone receptors (red) with GABA_B_ or mGluR2/3 receptors (green) in distinct hippocampal regions (CA1, CA3, DG, and hilus). Three background-corrected representative images were used to quantify each region in each group, and Pearson’s correlation coefficient was used as the co-localisation parameter^66^.

### Statistical analysis

For SE latency, serum analyses, and western blots, data were first transformed into Z-scores, after which the two-way ANOVA and Tukey’s post hoc tests were used. The statistical significance was set at *p* < 0.05. GraphPad Prism version 6.01 software (GraphPad Software, Inc., http://www.graphpad.com) was used in this study.

## Supplementary information

Supplementary Information.
