# Supplementary material for: Hormonal and biochemical changes in female Proechimys guyannensis, an animal model of resistance to pilocarpine-induced status epilepticus
Source: Sci Rep. 2020 Dec 2;10:20982. doi: 10.1038/s41598-020-77879-1 (PMC7710747; doi:10.1038/s41598-020-77879-1)
Supplement: Supplementary file 1 — Supplementary Information. [file 41598_2020_77879_MOESM1_ESM.pdf]

Hormonal and biochemical changes in female *Proechimys guyannensis*, an animal model of resistance to pilocarpine-induced status epilepticus

Viviam Sanabria[1+*], Simone Bittencourt[1+], Sandra Perosa[1], Tomás de la Rosa[1], Maria da Graça Naffah Mazzacoratti[1], Monica L. Andersen[2], Sergio Tufik[2], Esper A. Cavalheiro[1], Débora Amado[1]

[1]Department of Neurology and Neurosurgery, Universidade Federal de São Paulo (UNIFESP), Rua Pedro de Toledo, 669, São Paulo, SP, Brazil

[2]Department of Psychobiology, Universidade Federal de São Paulo (UNIFESP), Rua Botucatu, 826, São Paulo, SP, Brazil

[+]Authors with equal contribution.

***Correspondence and requests for materials should be addressed to:** Viviam Sanabria (email: vivisanabria124@gmail.com)

SUPPLEMENTARY INFORMATION

Supplementary Information A

Establishment of the dose–response relationship for SE induction with pilocarpine

To optimise the time-consuming experimental procedures and to decrease the mortality associated with the pilocarpine model, all experiments in female *Proechimys* were performed using the lithium–pilocarpine combination, as proposed by Glien et al.[1]. As marked intrastrain differences have been reported using this protocol in female rats from Europe[2], the protocol viability was tested in three female Wistar rats raised in the Brazilian climate.

This lithium–pilocarpine ramp-up protocol in female Wistar rats was useful for comparison with the conventional pilocarpine protocol, mainly in terms of the efficiency of the model in inducing spontaneous epileptic seizures, indicating sufficient damage to the central nervous system during SE. The dose of pilocarpine, latency to reach SE, and intensity of SE in between female Wistar rats and female *Proechimys*.

Spontaneous epileptic seizures observed in the chronic phase demonstrate the efficiency of the lithium–pilocarpine ramp-up protocol in inducing brain damage during SE. After 15 days, Wistars rats entered the chronic phase of the model. These animals were placed in the video room to observe the episodes for a period of 3 months (94 days). Considering stages 4 and 5 of the Racine scale, the Wistar rats showed an average of 32 spontaneous epileptic seizures in the first month, followed by an average of 49 and 50 seizures in the second and third months, respectively. The average duration of the seizures was 32.76±10.80 s. Based on the efficiency of the protocol used, the experiments in *Proechimys* were performed.

In female *Proechimys*, an initial dose of 10 mg/kg pilocarpine was administered, followed by 10 mg/kg every 30 min for up to five doses; however, no animal reached SE. The

protocol was repeated, as suggested by Glien et al.[1], with an initial pilocarpine dose of 40 mg/kg, followed by 20 mg/kg every 30 min for up to five doses. After the fifth dose, *Proechimys* were susceptible to pilocarpine but did not reach SE. We decided to continue the dose of 40 mg/kg every 30 min to reach SE, but at the seventh dose, *Proechimys* were no longer sensitised; surprisingly, they seemed completely recovered.

Considering that *Proechimys* were susceptible to six pilocarpine doses, we performed another experiment with initial doses of 250 mg/kg (as six doses of 40 mg/kg, totalling 240 mg/kg), followed by 250 mg/kg every 30 min for up to five doses. After establishing the dose–response relationship to induce SE with pilocarpine (250 mg/kg), we performed analyses at 30 and 90 min after SE onset. Figure 1 summarises all steps of the protocol that were followed to achieve SE in female *Proechimys*.

In conventional pilocarpine protocol, a dose of 320 mg/kg is commonly used to induce SE in male Wistar rats[3,4] without high mortality, and the same dose can induce SE in male *Proechimys*[5,6]. Susceptibility to SE induction is affected by the strain, age, and sex of animals. In the conventional pilocarpine protocol, male Wistar rats between 2 and 3 months of age require pilocarpine doses of 320 to 400 mg/kg to induce SE, although a dose of 400 mg/kg results in 100% mortality[7,8]. Older animals require a lower pilocarpine dose to develop SE without leading to death. This threshold particularly decreases beyond the age of 100 days[9]. In male *Proechimys* (2–3-month old), a dose of 320 mg/kg induced SE within 20–30 min after administration[5,6]; however, a slightly higher dose (350–380 mg/kg) led to death[6].

Supplementary Information B

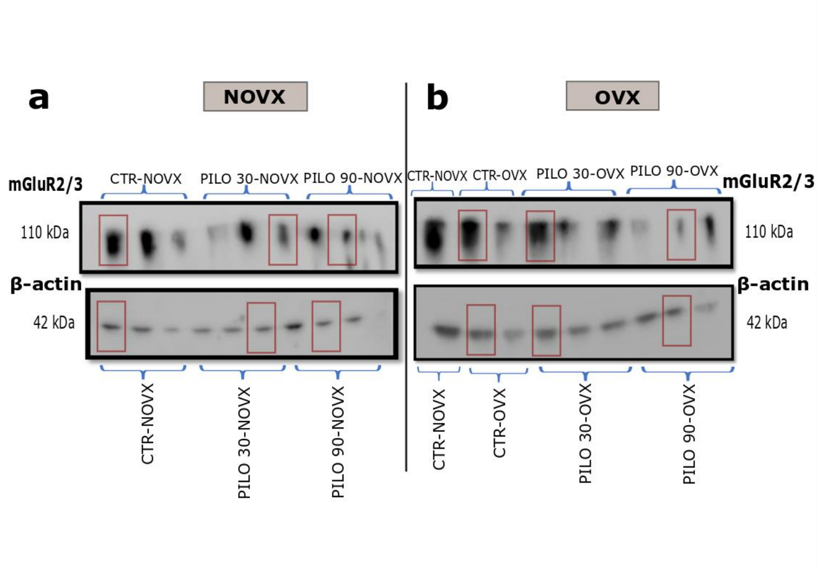

Figure S1. Full-length western blot of group II metabotropic glutamate receptor 2/3 (mGluR2/3) expression in the hippocampus of *Proechimys guyannensis* females with (a) or without (b) ovariectomy subjected to SE induction. β-Actin was used as a control for protein loading. Red boxes indicate the samples of interest as shown in main text.

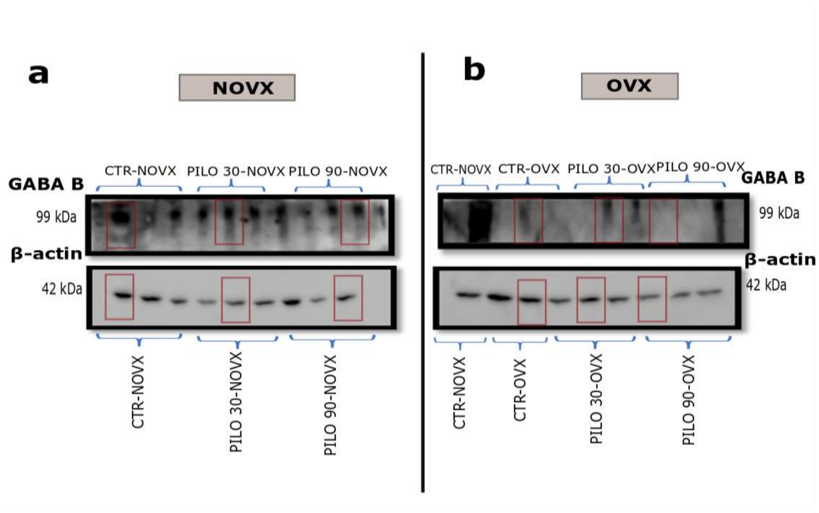

Figure S2. Full-length western blot of $GABA_B$ receptor ($GABA_B$) expression in the hippocampus of *Proechimys guyannensis* females with (a) or without (b) ovariectomy

subjected to SE induction. β-Actin was used as a control for protein loading. Red boxes indicate the samples of interest as shown in main text.

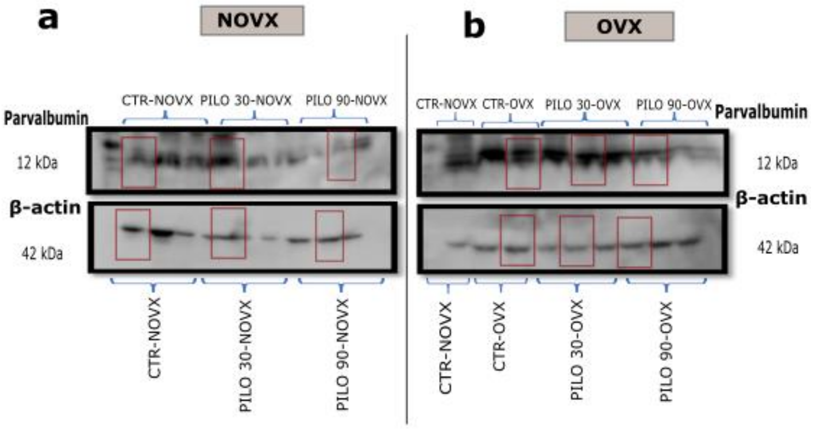

Figure S3. Full-length western blot of parvalbumin receptor expression in the hippocampus of *Proechimys guyannensis* females with (a) or without (b) ovariectomy subjected to SE induction. β-Actin was used as a control for protein loading. Red boxes indicate the samples of interest as shown in main text.

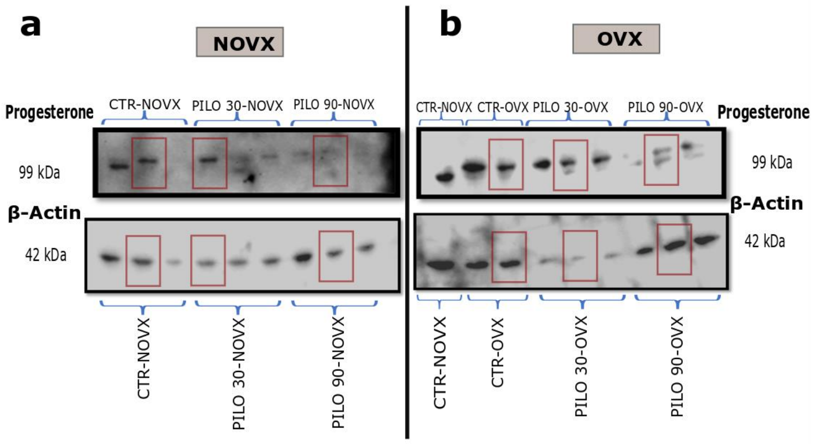

Figure S4. Full-length western blot of progesterone receptor expression in the hippocampus of *Proechimys guyannensis* females with (a) or without (b) ovariectomy subjected to SE

induction. β-Actin was used as a control for protein loading. Red boxes indicate the samples of interest as shown in main text.

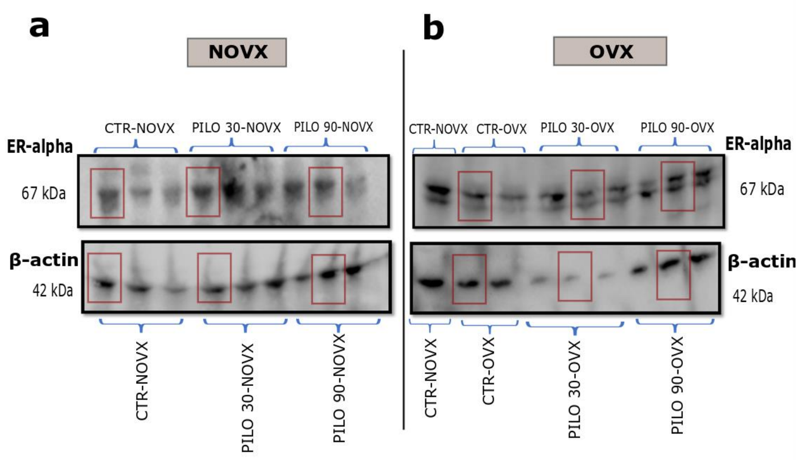

Figure S5. Full-length western blot of oestrogen receptor-alpha (ER-alpha) expression in the hippocampus of *Proechimys guyannensis* females with (a) or without (b) ovariectomy subjected to SE induction. β-Actin was used as a control for protein loading. Red boxes indicate the samples of interest as shown in main text.

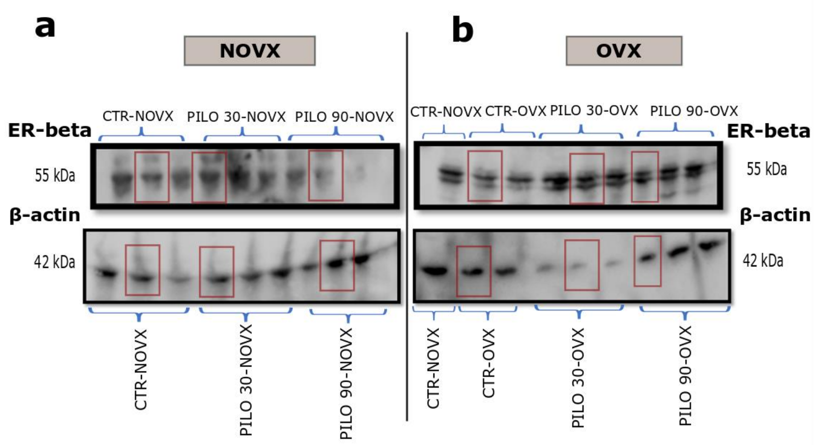

Figure S6. Full-length western blot of oestrogen receptor-beta (ER-beta) expression in the hippocampus of *Proechimys guyannensis* females with (a) or without (b) ovariectomy subjected to SE induction. β-Actin was used as a control for protein loading. Red boxes indicate the samples of interest as shown in main text.

References

1. Glien, M. *et al.* Repeated low-dose treatment of rats with pilocarpine: Low mortality but high proportion of rats developing epilepsy. *Epilepsy Res.* **46**, 111–119 (2001).
2. Brandt, C., Bankstahl, M., Töllner, K., Klee, R. & Löscher, W. The pilocarpine model of temporal lobe epilepsy: Marked intrastrain differences in female Sprague–Dawley rats and the effect of estrous cycle. *Epilepsy Behav.* **61**, 141–152 (2016).
3. Lemos, T. & Cavalheiro, E. A. Suppression of pilocarpine-induced status epilepticus and the late development of epilepsy in rats. *Exp. Brain Res.* **102**, 423–428 (1995).
4. Bittencourt, S., Covolan, L., Hamani, C., Longo, B. M. & Petralia, R. S. Replacement of asymmetric synaptic profiles in the molecular layer of dentate gyrus following cycloheximide in the pilocarpine model in rats. *Front. Psychiatry* **6**, 1–9 (2015).
5. Fabene, P. F., Correia, L., Carvalho, R. A., Cavalheiro, E. A. & Bentivoglio, M. The spiny rat Proechimys guyannensis as model of resistance to epilepsy: Chemical characterization of hippocampal cell populations and pilocarpine-induced changes. *Neuroscience* **104**, 979–1002 (2001).
6. Arida, R. M., Scorza, F. A., De Amorim Carvalho, R. & Cavalheiro, E. A. Proechimys guyannensis: An animal model of resistance to epilepsy. *Epilepsia* **46**, 189–197 (2005).
7. Mello, L. E. & Mendez-Otero, R. Expression of 9-O-acetylated gangliosides in the rat hippocampus. *Neurosci. Lett.* **213**, 17–20 (1996).
8. Turski, W. A. *et al.* Limbic seizures produced by pilocarpine in rats: Behavioural, electroencephalographic and neuropathological study. *Behav. Brain Res.* **9**, 315–335 (1983).
9. Patel, S., De Sarro, G. B. & Meldrum, S. Regulation of seizure threshold by excitatory amino acids in striatum and entopeduncular nucleus of rats. *Neuroscience* **27**, 837–850 (1988).
